# Expression of Connexin43 Stimulates Endothelial Angiogenesis Independently of Gap Junctional Communication In Vitro

**DOI:** 10.3390/ijms22147400

**Published:** 2021-07-09

**Authors:** Christoph Koepple, Zizi Zhou, Lena Huber, Matthias Schulte, Kjestine Schmidt, Torsten Gloe, Ulrich Kneser, Volker Jürgen Schmidt, Cor de Wit

**Affiliations:** 1 Department for Hand Surgery, Plastic Surgery and Reconstructive Surgery, BG Trauma Center Ludwigshafen, Heidelberg University, 67071 Ludwigshafen, Germany; zizi.zhou3@gmail.com (Z.Z.); lena.huber@hotmail.com (L.H.); matthias.schulte@rub.de (M.S.); ulrich.kneser@bgu-ludwigshafen.de (U.K.); 2 Institut für Physiologie, Universität zu Lübeck, 23562 Lübeck, Germany; kjestine.schmidt@uni-luebeck.de; 3 Deutsches Zentrum für Herz-Kreislauf-Forschung (DZHK) e.V. (German Center for Cardiovascular Research), 23562 Lübeck, Germany; 4 Physiology, Institute of Theoretical Medicine, Universität Augsburg, 86159 Augsburg, Germany; torsten.gloe@med.uni-augsburg.de; 5 Department for Plastic Surgery and Breast Surgery, Zealand University Hospital (SUH) Roskilde, Copenhagen University, 4000 Roskilde, Denmark

**Keywords:** gap junctions, connexins, endothelial tube formation, human umbilical vein endothelial cells, cellular migration

## Abstract

Connexins (Cx) form gap junctions (GJ) and allow for intercellular communication. However, these proteins also modulate gene expression, growth, and cell migration. The downregulation of Cx43 impairs endothelial cell migration and angiogenetic potential. Conversely, endothelial Cx43 expression is upregulated in an in vivo angiogenesis model relying on hemodynamic forces. We studied the effects of Cx43 expression on tube formation and proliferation in HUVECs and examined its dependency on GJ communication. Expectedly, intercellular communication assessed by dye transfer was linked to Cx43 expression levels in HUVECs and was sensitive to a GJ blockade by the Cx43 mimetic peptide Gap27. The proliferation of HUVECs was not affected by Cx43 overexpression using Cx43 cDNA transfection, siRNA-mediated knockdown of Cx43, or the inhibition of GJ compared to the controls (transfection of an empty vector, scrambled siRNA, and the solvent). In contrast, endothelial tube and sprout formation in HUVECs was minimized after Cx43 knockdown and significantly enhanced after Cx43 overexpression. This was not affected by a GJ blockade (Gap27). We conclude that Cx43 expression positively modulates the angiogenic potential of endothelial cells independent of GJ communication. Since proliferation remained unaffected, we suggest that Cx43 protein may modulate endothelial cell migration, thereby supporting angiogenesis. The modulation of Cx43 expression may represent an exploitable principle for angiogenesis induction in clinical therapy.

## 1. Introduction

Connexins are transmembrane proteins, which are the molecular bricks of intercellular channels (gap junctions). Six of these proteins assemble to form a hemichannel, which docks onto its counterpart from a neighboring cell to form a functional channel. These channels connect the cytosols of adjacent cells and therefore enable the exchange of ions, small metabolites, and signaling molecules between them [1]. They are critical for many physiological functions in the body, specifically those in which the activity of a multitude of cells has to be synchronized to ensure organ function [2]. Accordingly, gap junctions allow for the spread of signals in blood vessels, ensuring homogenous diameter changes along the length of the vessel during blood flow regulation [3,4,5]. The connexin family comprises 21 members in humans, which are named according to their molecular weight [6], and of these isoforms, four are expressed in vascular tissues (Cx43, Cx40, Cx37, and Cx45) [1,7].

In recent years, additional functions of connexins, specifically for Cx43, have been identified that are independent of gap junctional communication. For example, Cx43 has been demonstrated to modulate tumor growth, cell migration, and gene expression in a manner that does not rely on gap junctional communication or the formation of intercellular channels [8,9,10,11]. These functions are possibly related to the capability of, specifically, Cx43 to bind a multitude of proteins to its C-terminal domain that permits crosstalk between Cx43 and cytoskeletal as well as regulatory proteins [12,13]. Interacting proteins include transcription factors with consecutive modulation of gene expressions [14]. An impairment of migration during development throughout the body may also contribute to early postnatal death in mice with global Cx43 deletion [15]. Indeed, it has been long recognized that gap junction proteins act as multifaceted regulators in brain development [16]. Specifically, migration has been demonstrated to be dependent on Cx43 expression. It promotes adhesion between migrating cells, thereby enabling cell migration during brain development [17]. During the migratory process, neurons undergo a transient morphological transformation and Cx43 exerts an important control function in this process via signaling through other proteins [18].

Migration processes are essential not only for neuronal and embryonic development but also for adult maturation and wound healing as well as angiogenesis and vasculogenesis. Initial studies demonstrated that factors secreted by connexin-overexpressing cancer cells in vitro inhibited angiogenesis [19]. More importantly, Cx43 expression in nonneuronal cells enhanced their own migration. If only the carboxy tail of the protein was expressed, the positive modulation was still present, and conversely, migration was reduced in endothelial progenitor cells after Cx43 downregulation [20]. Similarly, downregulation of Cx43 in human aortic endothelial cells resulted in impaired proliferation, viability, and angiogenic potential. This was associated with an activation of c-jun N-terminal kinase (JNK) and its downstream target c-jun. Interestingly, the inhibition of JNK partially prevented these impairments, suggesting that the downregulation of Cx43 expression reduces the proangiogenic and proliferative potential of endothelial cells via the activation of the stress-activated protein kinase JNK [21]. Other investigations verified the detrimental effect of the downregulation of connexins in endothelial cells or endothelial progenitor cells on their angiogenic potential [22,23]. However, a possible dependency on gap junctional communication was not examined.

In a unique model of in vivo angiogenesis that is based on hemodynamic forces exerted on a grafted vessel implanted as an arteriovenous (AV) loop, angiogenesis can be examined without the need for the exogenous addition of angiogenic factors or their release from surrounding tissue [24]. Previously, we demonstrated that the enhancement of blood flow is decisive for the increased expression of endothelial Cx43 in a grafted vessel and for the concurrent initiation of vessel formation in this model [25,26]. Interestingly, endothelial Cx43 activation was also detected in AV loop grafts in humans [27]. Therefore, we hypothesize that the expression of Cx43 enhances the angiogenic potential of endothelial cells in a gap junction communication-independent manner. To test this hypothesis, we examined the effects of Cx43 up- and downregulation in human umbilical vein endothelial cells (HUVECs) in vitro on intercellular communication, proliferation, and angiogenesis. Furthermore, we assessed the efficacy of blocking gap junctional communication in these settings.

## 2. Results

### 2.1. Modulation of Cx43 Expression in HUVECs

Cx43 expression was assessed at the mRNA level using reverse transcription quantitative real-time PCR (RT-qPCR). Non-treated HUVECs were concurrently measured, and Cx43 expression after treatments is given as a relative expression (fold) normalized to Cx43 mRNA levels detected in non-treated HUVECs. The transfection of cells with Cx43 cDNA increased its expression 3.4-fold (3.44 ± 1.01-fold, *n* = 17, *p* < 0.05), whereas sham transfection (empty vector) did not modulate Cx43 expression (1.16 ± 0.30-fold, *n* = 15). Expectedly, treatment with siRNA markedly reduced the Cx43 expression in HUVECs (0.12 ± 0.05-fold, *n* = 4, *p* < 0.05).

### 2.2. Dye Transfer Is Modulated by Cx43 Expression and Blockage of Cx43-Dependent Coupling

**Figure 1:**
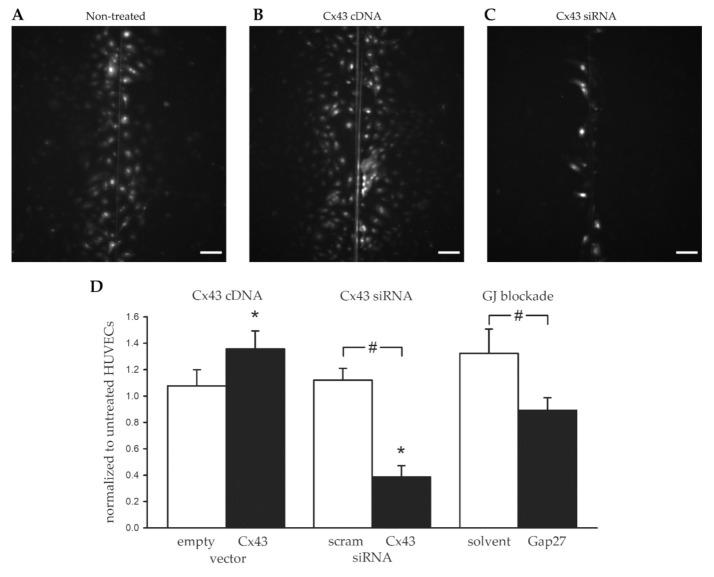
Dye transfer was modulated by Cx43 expression and GJ blockade. The representative images show dye transfer after scrape loading in non-treated controls (**A**), Cx43 cDNA transfected cells (**B**), and cells treated with Cx43 siRNA (**C**). Scale bar is 100 µm. The summary data in (**D**) are normalized to paired sample values obtained in non-treated HUVECs. The treatment groups are shown in black, and the respective controls are shown as white bars. Transfection with Cx43 cDNA enhanced the dye transfer, which was not found after transfection with an empty vector (left, *n* = 7). The scrambled siRNA (scram) had no effect, whereas Cx43 siRNA strongly decreased dye transfer (middle, *n* = 8). Dye transfer was significantly attenuated in cells treated with Gap27 compared to the solvent controls (right, *n* = 6). * *p* < 0.05 vs. non-treated HUVECs (paired *t*-test), # *p* < 0.05 vs. sham-treated control cells (paired *t*-test).

Dye transfer between cells after loading them with a fluorescent dye (lucifer yellow) by scraping reflects gap junctional communication because the dye is not transported through the membrane [28]. In the non-treated HUVECs, the dye covered an area of 0.81 ± 0.11 mm^2^. After transfection of the cells with Cx43 cDNA, the area covered by the dye increased to 1.05 ± 0.11 mm^2^ (*p* < 0.05 vs. non-treated, *n* = 7). In contrast, sham transfection (empty vector) did not change this area (0.85 ± 0.14 mm^2^; *p* = ns vs. non-treated). The downregulation of Cx43 using siRNA strongly reduced dye transfer. The dye covered only an area of 0.29 ± 0.05 mm^2^ (*n* = 7; 0.95 ± 0.18 mm^2^ in non-treated; *p* < 0.05). In contrast, treatment with scrambled siRNA did not modulate the dye area (0.99 ± 0.18 mm^2^). In cells treated with Gap27, which were all transfected with Cx43 cDNA, the dye area amounted to 0.92 ± 0.20 mm^2^ (*n* = 6), which was significantly reduced compared to cells transfected with Cx43 cDNA and treated only with the solvent of Gap27 (1.31 ± 0.27 mm^2^; *p* < 0.05). These data are illustrated by representative images and summarized as values normalized to the respective non-treated HUVECS in Figure 1.

### 2.3. Cell Proliferation Remained Unaltered by Modulation of Functional Cx43

**Figure 2:**
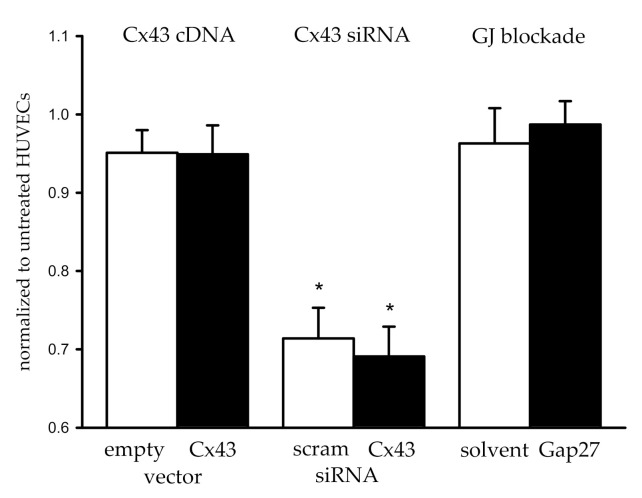
Proliferation assessed by BrdU incorporation remained unaltered by modulation of Cx43. The absorption values after BrdU incorporation are normalized to paired sample values obtained in non-treated HUVECs. The treatment groups are shown in black, and the respective controls are shown as white bars. Proliferation was not different after transfection with Cx43 cDNA (*n* = 36), Cx43 siRNA (*n* = 33), or Gap27 (*n* = 54) compared to the respective sham treatment group. Treatment with siRNA decreased proliferation markedly but to a similar degree in the scrambled (scram) and Cx43 siRNA group. * *p* < 0.05 vs. non-treated HUVECs (paired *t*-test), differences between treatment and sham-treated groups were not detected (paired *t*-test).

Cell proliferation was quantified using the Bromdesoxyuridine (BrdU) incorporation assay in the three treatment groups. The absorption due to incorporated BrdU in the respective groups was normalized to the absorption measured in non-treated HUVECs, which were examined in parallel on the same days. In cells transfected with Cx43 cDNA as well as those transfected with an empty vector (sham), BrdU incorporation tended to be slightly lower than in non-treated cells (normalized to non-treated: 0.93 ± 0.02 and 0.95 ± 0.03, respectively; *n* = 36). However, both groups (Cx43 cDNA, empty vector) were not different from each other (Figure 2). In cells transfected with siRNA, BrdU incorporation was strongly attenuated (≈0.70 of non-treated, *n* = 33). This attenuation was similarly observed in cells treated with Cx43 siRNA and scrambled siRNA; thus, both groups were not different from each other (Figure 2), indicating nonspecific inhibition of proliferation due to the siRNA transfection protocol. BrdU incorporation was also slightly reduced in cells treated with Gap27 or its solvent (and both transfected with Cx43 cDNA, *n* = 54) compared to non-treated HUVECs examined in parallel. Again, these two groups (treatment with Gap27 or its solvent) were not different from each other (Figure 2).

### 2.4. Enhancement of Tube Formation by Cx43 Expression Remained Unaffected by Gap27

**Figure 3:**
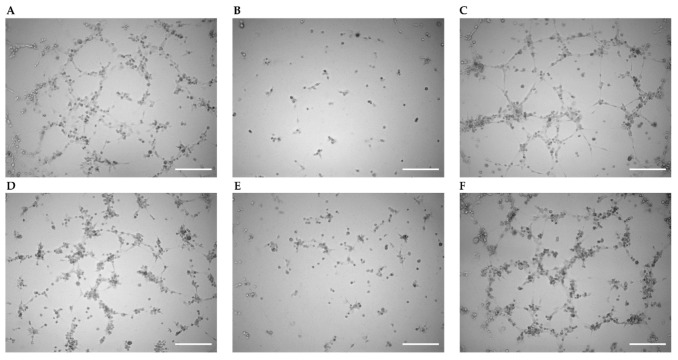
Angiogenesis assay in different treatment groups. Representative images obtained 6 h after the start of the tube formation assay are shown for non-treated HUVECs (**A**), cells transfected with an empty vector (**B**) or with Cx43 cDNA (**C**), cells treated with scrambled siRNA (**D**) or Cx43 siRNA (**E**), and cells transfected with Cx43 cDNA followed by Gap27 application (**F**). Scale bar is 250 µm.

**Figure 4:**
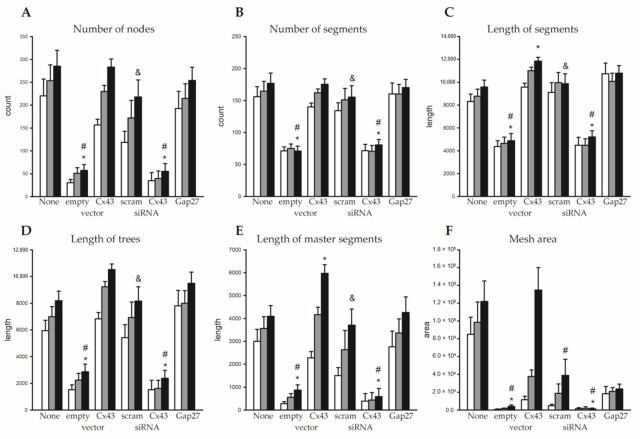
Angiogenic potential of HUVECs was modulated by Cx43 expression but did not depend on GJ communication. Quantitative analysis of tube formation assays in different treatment groups 2 h (white bars), 4 h (grey bars), and 6 h (black bars) after the start of the assay. Number of nodes (**A**) and segments (**B**); total sum of the length of segments (**C**), trees (**D**), or master segments (**E**); as well as the area of meshes (**F**) are depicted. In comparison to non-treated HUVECs (*n* = 38), transfection of the empty vector (*n* = 23) reduced all parameters of angiogenesis, which was overcome if Cx43 cDNA (*n* = 23) was transfected. Treatment with Cx43 siRNA (*n* = 19) likewise abrogated the angiogenic potential, which was not observed for scrambled siRNA (scram, *n* = 10). Treatment with Gap27 after transfection of Cx43 cDNA (*n* = 11) was without effect on angiogenesis. Statistical analysis was only performed at timepoint 6 h. * *p* < 0.05 vs. non-treated HUVECs, # *p* < 0.05 vs. Cx43 cDNA, & *p* < 0.05 vs. Cx43 siRNA (only analyzed for scrambled siRNA), one-way analysis of variance (ANOVA) followed by Bonferroni multiple-comparison test.

Angiogenesis was studied via an assessment of tube formation in HUVECs in different treatment groups in parallel including a non-treated control group. Images were taken 2, 4, and 6 h after the cells were applied into the wells of slides to initiate angiogenesis. Representative images obtained after 6 h are shown in Figure 3 for all groups, i.e., non-treated, sham vector, Cx43 cDNA, scrambled siRNA, Cx43 siRNA, and cells treated with Cx43 cDNA followed by Gap27 application. Endothelial tubes were formed in all groups except in those transfected with the sham vector or Cx43 siRNA. In the quantitative analysis nodes, segments (isolated elements and branches), trees (segments and branches), master segments (elements connecting junctions), and meshes (area enclosed by segments) were identified, and their number and length increased with time in non-treated HUVECs (Figure 4).

In cells transfected with the empty sham vector, all of these parameters were strongly reduced compared to non-treated controls. Statistical analysis was performed only after 6 h, and at this time point, all of these parameters were significantly reduced compared to non-treated cells (*p* < 0.05, Figure 4). The attenuation is most likely due to the transfection protocol that abrogates the angiogenic potential of HUVECs. Such an attenuation was, however, not observed if Cx43 cDNA was transfected. While most parameters were not different from non-treated cells, some (master segment length and segment length) were significantly enhanced compared to non-treated cells (*p* < 0.05). This suggests that the attenuating effect of the transfection procedure is offset and overcome by Cx43 cDNA transfection. Treatment with scrambled siRNA did not reduce the parameters of tube formation compared to non-treated cells. In marked contrast, Cx43 siRNA nearly abrogated angiogenesis and all parameters were significantly reduced compared to non-treated cells (*p* < 0.05, Figure 4). Blocking Cx43-dependent gap junctional communication using Gap27 (after Cx43 cDNA transfection) did not affect the tube formation parameters compared to non-treated cells or compared to cells only transfected with Cx43 cDNA (Figure 4).

## 3. Discussion

The present study demonstrates that the expression of Cx43 modulates the angiogenic potential of endothelial cells in vitro. Transfection of HUVECs with Cx43 cDNA enhances and specific downregulation of Cx43 expression by siRNA strongly attenuates the angiogenesis examined in a tube formation assay. This proangiogenic effect of Cx43 was not blocked by interference with the Cx43-dependent gap junctional communication, and we therefore conclude that the observed positive modulatory effect of Cx43 on angiogenesis is independent of its well-known function of enabling intercellular communication. In an in vivo model of angiogenesis based on enhanced blood flow in a grafted vessel (arteriovenous shunt) [24], we previously demonstrated that the enhancement of blood flow is decisive in the initiation of the ensuing vessel formation [26]. This angiogenesis is accompanied by an increased expression of endothelial Cx43 in the grafted vessel [25]. The angiogenic potential of a such an arteriovenous loop acting as a vascular carrier can be utilized in plastic surgery to promote axial neovascularization and, thus, to prime a specific tissue unit for transfer using microsurgical techniques once intrinsic vascularization has been achieved [29,30]. The present data suggest that Cx43 indeed may have a role in such a setting although HUVECs were studied in vitro without flow.

The upregulation of Cx43 by transfection of Cx43 cDNA enhanced dye transfer in HUVECs, which indicates that, after transfection, Cx43 proteins are also implemented at a higher level into the membrane and form functional gap junctions. Importantly, this effect could be blocked by the connexin-mimetic peptide Gap27, which specifically blocks Cx43-dependent communication [31]. Conversely, the downregulation by Cx43 siRNA not only reduced Cx43 at the mRNA level but also strongly abrogated dye transfer, suggesting that Cx43 is an important connexin providing gap junctional communication in HUVECs, which was also demonstrated previously and is in line with expression data [22,32]. Thus, the experimental maneuvers induced functional responses on the cellular level with regard to gap junctional communication, as expected.

Other investigators have demonstrated that angiogenesis is reduced after the knockdown of Cx43 using siRNA in HUVECs [22], in human aortic endothelial cells [21], as well as in endothelial progenitor cells, which was associated with a lack of their therapeutic potential in hind limb ischemia in mice [23]. Similarly, the present data demonstrate a remarkable impairment of angiogenic activity in tube formation assays after treatment with Cx43 siRNA. This effect was specific since scrambled siRNA did not attenuate angiogenic potential compared to non-treated controls. Unexpectedly, the transfection of an empty vector to transmit cDNA severely affected the angiogenic potential of HUVECs, thus minimizing tube formation after 6 h, which is obviously related to the transfection protocol and/or the incorporation of the empty vector. However, implementing Cx43 cDNA into the vector completely restored the angiogenic potential of HUVECs. Some of the analyzed parameters, such as the length of segments or of master segments, were even enhanced compared to non-treated cells, demonstrating a profound positive modulatory effect of Cx43 protein on angiogenesis. Most interestingly, this enhancement was not attenuated by Gap27, which we verified impaired dye transfer in our experiments. Therefore, we conclude that the expression of Cx43 protein enhances the angiogenic potential of endothelial cells in a manner that is independent of gap junctional communication. Thus, our present study extends previous reports that examined only the effect of Cx43 knockdown, and furthermore, we provide conclusive indication that the effect of Cx43 is not dependent on the well-known function of connexins allowing gap junctional communication.

The downregulation of Cx43 was suggested to be associated with decreased proliferation and survival of endothelial progenitor cells, which was possibly related to the activation of the c-jun N-terminal kinase (JNK) [23]. Contradictory findings were obtained in other cells. The expression of the carboxy tail of Cx43, which was also found to be translocated into the nucleus, inhibited growth and proliferation [33,34]. Similarly, Cx43 carrying single mutations in the extracellular loop diminished cell proliferation supporting a gap junction-independent effect of Cx43 on cell growth [35]. In the present experiments, we did not find a significant effect of modulating the expression of Cx43 on proliferation in HUVECs as assayed by BrdU incorporation into the DNA. Only the treatment with siRNA exerted a drastic reduction in proliferation compared to non-treated cells. However, such an attenuation of cell proliferation was similarly observed in the siRNA control group (scrambled) and, thus, is rather nonspecific and most likely related to the siRNA transfection protocol. Likewise, the blockade of gap junctional communication through Cx43 was without effect. This suggests that the positive effect on angiogenesis is not exerted by modulating cell proliferation.

It is intriguing to speculate that the gap junction-independent proangiogenic effects of Cx43 may be due to an effect on cell migration, which was reported previously for neuronal [16,17] and other cells including endothelial cells [1,9,20]. The modulation of cell migration was p38 MAP kinase-dependent and may specifically involve the functional C-terminus of Cx43 [20]. Others have identified an interaction of Cx43 with zonula occludens-1 (ZO-1) by which varying levels of Cx43 regulated F-actin cytoskeletal architecture and modulated wound healing in endothelial cells [36]. Although these data suggest that Cx43 physically interacts with other proteins within the cell to modulate the cytoskeleton or to initiate signaling within a signalosome to result in the phosphorylation of target proteins, it should be considered that Cx43 assembles in the membrane, forms hemichannels, and releases mediators such as ATP [37] to modulate cell migration and angiogenesis. However, Gap27 not only blocked gap junctional communication but also inhibited Cx43 hemichannel opening in cardiomyocytes and brain endothelial cells [38,39]. Other connexins have also been shown to affect angiogenesis. Cx32 enhanced the angiogenic potential of HUVECs, and conversely, Cx32 deficiency impaired vascular sprouting and cell migration [40]. Endothelial cells also express abundant amounts of Cx40 and Cx37 [4]. Interestingly, these connexins exhibited opposite effects on angiogenesis and arteriogenesis. Targeting Cx40 expression or function reduced angiogenesis in the developing mouse retina [41] and reduced tumoral angiogenesis and its growth [42], while the deletion of Cx37 exerted opposite effects. In a hindlimb ischemia model, Cx40 deletion deteriorated limb perfusion after ischemia induction whereas Cx37 ablation enhanced recovery [43]. These divergent results may be explained by the role of Cx40 in arteriogenesis [44], whereas Cx37 exerts suppressive effects on cellular proliferation [45]. It will be interesting to explore the gap junctional-independent effects of connexins with respect to angiogenesis in other models of disease.

In conclusion, we demonstrated that the expression level of Cx43 in endothelial cells positively modulates their angiogenetic potential. This effect is most likely independent of gap junctional communication. These data add to the current understanding of endothelial tube formation and may provide a mechanistic explanation for the previously described initiation of vessel formation from a grafted vessel in a model of angiogenesis that relies on increases in blood flow in vivo. The modulation of Cx43 expression may be an exploitable principle for angiogenesis induction in clinical therapy.

## 4. Materials and Methods

### 4.1. Experimental Design

We performed tube formation assays, RT-qPCR, proliferation assays using Bromodeoxyuridine (BrdU), and scrape loading/dye transfer experiments in human umbilical vein endothelial cells (HUVECs). Untreated HUVECS as well as HUVECs transfected with Cx43 cDNA using a vector (pcDNA3.2-Cx43-HA) or with siRNA directed against Cx43 were examined. An empty pcDNA3.1 vector and scrambled siRNA were used as controls. The connexin-mimetic peptide Gap27 [46] (Sigma-Aldrich, Darmstadt, Germany) was applied to block Cx43-dependent gap junctional communication (GJ blockade). Importantly, for each experiment, in these three experimental groups (Cx43 transfection, Cx43 siRNA, and GJ blockade using Gap27), the respective sham treatment experiment (pcDNA3.1, scrambled siRNA, and DMSO as a solvent for Gap27) was performed in parallel (together with non-treated HUVECs) on the same day in cells obtained from the same flasks in order to perform an experimental design that resulted in paired samples unless otherwise stated.

### 4.2. Preparation and Culture of HUVECs

Endothelial cells were extracted from human umbilical cord veins by collagenase digestion and cultured as described previously [47]. In brief, umbilical cords stored in a sterile container at 4 °C were obtained once weekly from a local hospital (procedure approved by the local ethic committee). Human umbilical cord vein endothelial cells (HUVECs) from the cord lumen were extracted following collagenase I (100 U/mL; Sigma-Aldrich, Darmstadt, Germany) digestion by perfusion with Hanks’ Balanced Salt solution (HBSS, Merck KGaA, Darmstadt, Germany). The purity of the extracted HUVECs was verified to be greater than 98% using anti-CD31 antibodies by FACS analysis (data not shown). Pooled HUVECs were grown in culture flasks or wells that were coated with 0.1% gelatine solution. M199 medium (Gibco, Schwerte, Germany) supplemented with 10% fetal bovine serum (Capricorn, Ebsdorfergrund, Germany), large vessel endothelial supplement (Gibco, Carlsbad, CA, USA), penicillin and streptomycin (100 U/mL, Gibco, Waltham, MA, USA), and heparin (10 U/mL, Sigma-Aldrich, St. Louis, MO, USA) were used for the culture. The same medium without added antibiotics was used for transfection experiments. HUVECs were cultured at 37 °C in humidified air supplemented with 5% CO_2_. HUVECs were seeded in gelatine-coated six-well plates at a density of 2 × 10^5^ cells/well. For proliferation experiments (BrdU assays), 10^4^ cells/well were seeded in gelatine-coated 96-well plates.

### 4.3. Plasmid Constructs, Transfection of Plasmid-cDNA and SiRNA, and Treatment with Gap27

The human Cx43 clone pcDNA3.2-Cx43-HA was obtained from the open plasmid repository (Addgene, Cambridge, MA, USA, plasmid no. 49851), and pcDNA3.1 (Invitrogen, Schwerte, Germany) was used as the empty control vector. Transfection-grade plasmid DNA was purified from *Escherichia coli* DH5α after transformation (Thermo Fisher, Schwerte, Germany) using a Plasmid Plus Maxi Kit (Qiagen, Hilden, Germany) according to the manufacturer’s protocol. All DNA constructs were confirmed by sequencing (SeqLab, Goettingen, Germany). Human Cx43-siRNA (sc-29276) as well as control siRNA (sc-37007) were purchased from Santa Cruz Biotechnology (Dallas, TX, USA).

For the protein knockdown experiments, cells were grown on a six-well plate and transfected with 30 pmol small interfering RNA (siRNA) per well using Lipofectamine RNAiMAX (Invitrogen, Schwerte, Germany) according to the manufacturer’s recommendations in an antibiotic-free medium and incubated overnight. For cDNA transfection, the cells in each well were transfected with 1.0 µg of Cx43 cDNA (pcDNA3.2-Cx43-HA) or an empty pcDNA3.1 vector in antibiotic-free medium using the GenJet™ transfection reagent (SignaGen Laboratories, Rockville, MD, USA) according to the manufacturer’s protocol and incubated overnight. After transfection, the HUVECs were washed and a cell culture medium was added before the experiments (tube formation, dye transfer, RT-qPCR, or proliferation assay) started. In all experiments using Gap27, the HUVECs were transfected with Cx43 cDNA. In these experiments, the HUVECs were washed similarly with phosphate-buffered saline (PBS) after the Cx43 cDNA transfection protocol. Thereafter, the culture medium as well as Gap27 diluted in medium (from a stock solution prepared in sterile DMSO at 50 mmol/L and stored at −20 °C until use) was added to achieve a final concentration of 100 µmol/L Gap27. Herein, the HUVECs were incubated for 24 h and washed again in PBS before the continuation of the experiments. An equivalent amount of DMSO was added in the respective control experiments and incubated for a similar time period (24 h).

### 4.4. Tube Formation Assay

This assay was performed in different treatment groups in parallel including a non-treated control group resulting in non-paired samples. The treatment groups were transfected with pcDNA3.2-Cx43-HA or pcDNA3.1 (empty control vector), Cx43 siRNA or scrambled siRNA, as well as a group of cells treated with Cx43 cDNA followed by Gap27 application. Trypsinized cells were resuspended in culture medium and counted after trypan blue staining. Specific angiogenesis plates (µ-Slide™, Ibidi, Martinsried, Germany) were prepared according to the manufacturer’s recommendations and coated with matrix (10 µL of Cultrex™ reduced growth factor basement membrane matrix, Trevigen, Gaithersburg, MD, USA) on the day before the HUVECs were added. Five thousand cells dissolved in 50 µL culture medium were applied into each well of the µ-slide™ and incubated at 37 °C in humidified air supplemented with 5% CO_2_. The cells were imaged after incubation periods of 2, 4, and 6 h using a fully motorized microscope equipped with CCD camera (BZ-9000, Keyence, Neu-Isenburg, Germany) using the 10× objective. Bright field images with a resolution of 1360 × 1024 pixels were acquired using an exposure time of 1/1900 s. The images were analyzed by ImageJ on a PC. First, the noise was reduced without removing the edges or details by anisotropic diffusion [48]. Then, capillary tube formation of the HUVECs was quantified using the ImageJ plugin “Angiogenesis Analyser” [49]. This software allows for quantification of a number of different parameters. We evaluated the number of nodes (defined as a pixel with more than three neighbors, corresponding to a bifurcation), the number and sum of the length of segments, the isolated elements and branches, the sum of the length of trees (composed of segments and branches), the sum of the length of master segments (defined as elements connecting junctions formed by a group of nodes), and the area of meshes in the network (defined as the area enclosed by segments).

### 4.5. Quantitative Real-Time PCR

Total RNA extraction from HUVECs was performed using innuPREP RNA Mini Kit 2.0 (Analytik Jena, Jena, Germany). The total RNA (250 ng) was reverse transcribed using M-MuLV reverse transcriptase in the presence of murine RNase-Inhibitor (New England Biolabs, Frankfurt, Germany) with random hexamers and dNTPs (Thermo Fisher Scientific, Schwerte, Germany) according to the manufacturer’s protocol. RT-qPCR was performed using 1 μL of acquired cDNA for Cx43 detection or 1 µL of diluted cDNA (1:500) for L28 detection. A total of 5 µL of the 2xqPCRBIO SyGreen Mix (Nippon Genetics Europe GmbH, Düren, Germany) and 0.4 µL of the following primers (10 pmol/µL) were added:
Cx43:forward 5′-CTGAGTGCCTGAACTTGCCT-3′
reverse 5′-CCTGGGCACCACTCTTTTGC-3′L28:forward 5′-ATGGTCGTGCGGAACTGCT-3′
reverse 5′-TTGTAGCGGAAGGAATTGCG-3′

The relative levels of human Cx43 mRNA were normalized to the corresponding levels of human L28 and analyzed as described by Livak and Schmittgen using the 2^−ΔΔCT^ method [50].

### 4.6. Scrape Loading/Dye Transfer Assay

Intercellular communication via gap junctions was characterized using dye transfer after scrape loading, as described before [28]. In brief, confluent HUVEC monolayers in six-well plates were used. Three cuts were applied with a surgical blade in each well after washing with PBS containing Ca^2+^ and Mg^2+^ three times. Thereafter, cells were loaded with lucifer yellow dye (Sigma-Aldrich, Darmstadt, Germany) by incubation of the cells in a warmed dye solution (1 mg/mL) at room temperature for 5 min in the dark. After this period, the dye solution was rigorously washed away and the HUVECs were fixed using 10% formalin. Fluorescence and bright field images of the individual scratches were immediately acquired thereafter using the motorized inverted fluorescence microscope described above. Images were taken using a 10× objective and exposure times of 1/7 or 1/1900 s and stored on a hard disk at a resolution of 1360 × 1024 pixels for analysis later. The area covered by stained cells was determined as the readout of dye diffusion through gap junctions using ImageJ. Hitherto, the images were converted to black-and-white using a threshold function and the area was measured after calibration.

### 4.7. Bromodeoxyuridine (BrdU) Incorporation Assay

Cell proliferation was measured using a standardized ELISA-based BrdU incorporation assay kit (Merck, Darmstadt, Germany) according to the manufacturer’s instructions; 10^4^ cells/mL were seeded in 96-well plates and transfected or treated as described above using adjusted amounts of pcDNA3.2-Cx43-HA (0.125 µg) or siRNA (5 pmol). Absorbance was measured at 450 and 595 nm using a microplate reader (Multiskan FC Microplate Reader, Thermo Scientific, Schwerte, Germany). The experiments were carried out in triplicates, and the results were averaged and taken as a single observation.

### 4.8. Data Analysis

Statistical analysis was performed using STATA (Stata Corporation, College Station, TX, USA). The data are presented as a mean ± standard error of mean (mean ± S.E.M.). Paired samples were analyzed by the paired *t*-test. Unpaired data (tube formation assay) were compared using one-way analysis of variance (ANOVA) followed by Bonferroni multiple-comparison test. Differences were considered significant at a corrected error probability of *p* < 0.05.

## Data Availability

The data presented in this study are available on request from the corresponding author.

## References

[1] Pohl U. (2020). Connexins: Key Players in the Control of Vascular Plasticity and Function. Physiol. Rev..

[2] Leybaert L., Lampe P.D., Dhein S., Kwak B., Ferdinandy P., Beyer E., Laird D.W., Naus C.C., Green C.R., Schulz R. (2017). Connexins in Cardiovascular and Neurovascular Health and Disease: Pharmacological Implications. Pharmacol. Rev..

[3] Schmidt V.J., Wölfle S.E., Boettcher M., De Wit C. (2008). Gap junctions synchronize vascular tone within the microcirculation. Pharmacol. Rep..

[4] Jobs A., Schmidt K., Schmidt V.J., Lübkemeier I., Van Veen T.A., Kurtz A., Willecke K., De Wit C. (2012). Defective Cx40 Maintains Cx37 Expression but Intact Cx40 Is Crucial for Conducted Dilations Irrespective of Hypertension. Hypertension.

[5] Pogoda K., Kameritsch P., Mannell H., Pohl U. (2019). Connexins in the control of vasomotor function. Acta Physiol..

[6] Söhl G., Willecke K. (2004). Gap junctions and the connexin protein family. Cardiovasc. Res..

[7] Schmidt V.J., Jobs A., von Maltzahn J., Wörsdörfer P., Willecke K., de Wit C. (2012). Connexin45 is expressed in vascular smooth muscle but its function remains elusive. PLoS ONE.

[8] Jiang J.X., Gu S. (2005). Gap junction- and hemichannel-independent actions of connexins. Biochim. Biophys. Acta BBA Biomembr..

[9] Kameritsch P., Pogoda K., Pohl U. (2012). Channel-independent influence of connexin 43 on cell migration. Biochim. Biophys. Acta BBA Biomembr..

[10] Zhou J.Z., Jiang J.X. (2014). Gap junction and hemichannel-independent actions of connexins on cell and tissue functions—An update. FEBS Lett..

[11] Martins-Marques T., Ribeiro-Rodrigues T., Batista-Almeida D., Aasen T., Kwak B.R., Girao H. (2019). Biological Functions of Connexin43 Beyond Intercellular Communication. Trends Cell Biol..

[12] Matsuuchi L., Naus C.C. (2013). Gap junction proteins on the move: Connexins, the cytoskeleton and migration. Biochim. Biophys. Acta BBA Biomembr..

[13] Leithe E., Mesnil M., Aasen T. (2018). The connexin 43 C-terminus: A tail of many tales. Biochim. Biophys. Acta BBA Biomembr..

[14] Kotini M., Barriga E.H., Leslie J., Gentzel M., Rauschenberger V., Schambony A., Mayor R. (2018). Gap junction protein Connexin-43 is a direct transcriptional regulator of N-cadherin in vivo. Nat. Commun..

[15] Reaume A., De Sousa P., Kulkarni S., Langille B., Zhu D., Davies T., Juneja S., Kidder G., Rossant J. (1995). Cardiac malformation in neonatal mice lacking connexin43. Science.

[16] Elias L.A., Kriegstein A.R. (2008). Gap junctions: Multifaceted regulators of embryonic cortical development. Trends Neurosci..

[17] Elias L.A.B., Wang D.D., Kriegstein A.R. (2007). Gap junction adhesion is necessary for radial migration in the neocortex. Nature.

[18] Liu X., Sun L., Torii M., Rakic P. (2012). Connexin 43 controls the multipolar phase of neuronal migration to the cerebral cortex. Proc. Natl. Acad. Sci. USA.

[19] McLachlan E., Shao Q., Wang H.-L., Langlois S., Laird D.W. (2006). Connexins Act as Tumor Suppressors in Three-dimensional Mammary Cell Organoids by Regulating Differentiation and Angiogenesis. Cancer Res..

[20] Behrens J., Kameritsch P., Wallner S., Pohl U., Pogoda K. (2010). The carboxyl tail of Cx43 augments p38 mediated cell migration in a gap junction-independent manner. Eur. J. Cell Biol..

[21] Wang H.H., Kung C.I., Tseng Y.Y., Lin Y.C., Chen C.H., Tsai C.H., Yeh H.I. (2008). Activation of endothelial cells to pathological status by down-regulation of connexin43. Cardiovasc. Res..

[22] Gärtner C., Ziegelhöffer B., Kostelka M., Stepan H., Mohr F.-W., Dhein S. (2012). Knockdown of endothelial connexins impairs angiogenesis. Pharmacol. Res..

[23] Wang H.-H., Su C.-H., Wu Y.-J., Li J.-Y., Tseng Y.-M., Lin Y.-C., Hsieh C.-L., Tsai C.-H., Yeh H.-I. (2013). Reduction of connexin43 in human endothelial progenitor cells impairs the angiogenic potential. Angiogenesis.

[24] Polykandriotis E., Tjiawi J., Euler S., Arkudas A., Hess A., Brune K., Greil P., Lametschwandtner A., Horch R.E., Kneser U. (2008). The venous graft as an effector of early angiogenesis in a fibrin matrix. Microvasc. Res..

[25] Schmidt V.J., Hilgert J.G., Covi J.M., Weiß C., Wietbrock J.O., De Wit C., Horch R.E., Kneser U. (2013). High Flow Conditions Increase Connexin43 Expression in a Rat Arteriovenous and Angioinductive Loop Model. PLoS ONE.

[26] Schmidt V.J., Hilgert J.G., Covi J.M., Leibig N., Wietbrock J.O., Arkudas A., Polykandriotis E., de Wit C., Horch R.E., Kneser U. (2015). Flow Increase Is Decisive to Initiate Angiogenesis in Veins Exposed to Altered Hemodynamics. PLoS ONE.

[27] Henn D., Abu-Halima M., Wermke D., Falkner F., Thomas B., Kopple C., Ludwig N., Schulte M., Brockmann M.A., Kim Y.J. (2019). MicroRNA-regulated pathways of flow-stimulated an-giogenesis and vascular remodeling in vivo. J. Transl. Med..

[28] Babica P., Sovadinova I., Upham B.L. (2016). Scrape Loading/Dye Transfer Assay. Methods Mol. Biol..

[29] Polykandriotis E., Arkudas A., Beier J.P., Hess A., Greil P., Papadopoulos T., Kopp J., Bach A.D., Horch R.E., Kneser U. (2007). Intrinsic Axial Vascularization of an Osteoconductive Bone Matrix by Means of an Arteriovenous Vascular Bundle. Plast. Reconstr. Surg..

[30] Arkudas A., Beier J.P., Heidner K., Tjiawi J., Polykandriotis E., Srour S., Sturzl M., Horch R.E., Kneser U. (2007). Axial prevascularization of porous matrices using an arteriovenous loop promotes survival and differentiation of transplanted autologous osteoblasts. Tissue Eng..

[31] Cotter M.L., Boitano S., Lampe P.D., Solan J.L., Vagner J., Ek-Vitorin J.F., Burt J.M. (2019). The lipidated connexin mimetic peptide SRPTEKT-Hdc is a potent inhibitor of Cx43 channels with specificity for the pS368 phospho-isoform. Am. J. Physiol. Cell Physiol..

[32] Kameritsch P., Khandoga N., Nagel W., Hundhausen C., Lidington D., Pohl U. (2005). Nitric oxide specifically reduces the permeability of Cx37-containing gap junctions to small molecules. J. Cell. Physiol..

[33] Dang X., Doble B.W., Kardami E. (2003). The carboxy-tail of connexin-43 localizes to the nucleus and inhibits cell growth. Mol. Cell. Biochem..

[34] Moorby C., Patel M. (2001). Dual Functions for Connexins: Cx43 Regulates Growth Independently of Gap Junction Formation. Exp. Cell Res..

[35] Olbina G., Eckhart W. (2003). Mutations in the second extracellular region of connexin 43 prevent localization to the plasma membrane, but do not affect its ability to suppress cell growth. Mol. Cancer Res..

[36] Chen C.-H., Mayo J.N., Gourdie R.G., Johnstone S.R., Isakson B.E., Bearden S.E. (2015). The connexin 43/ZO-1 complex regulates cerebral endothelial F-actin architecture and migration. Am. J. Physiol. Physiol..

[37] Wang N., De Bock M., Decrock E., Bol M., Gadicherla A., Vinken M., Rogiers V., Bukauskas F.F., Bultynck G., Leybaert L. (2013). Paracrine signaling through plasma membrane hemichannels. Biochim. Biophys. Acta BBA Biomembr..

[38] Wang N., De Bock M., Antoons G., Gadicherla A.K., Bol M., Decrock E., Evans W.H., Sipido K.R., Bukauskas F.F., Ley-baert L. (2012). Connexin mimetic peptides inhibit Cx43 hemichannel opening triggered by voltage and intracellular Ca^2+^ elevation. Basic Res. Cardiol..

[39] De Bock M., Culot M., Wang N., Bol M., Decrock E., De Vuyst E., Da Costa A., Dauwe I., Vinken M., Simon A.M. (2011). Connexin Channels Provide a Target to Manipulate Brain Endothelial Calcium Dynamics and Blood—Brain Barrier Permeability. Br. J. Pharmacol..

[40] Okamoto T., Akita N., Kawamoto E., Hayashi T., Suzuki K., Shimaoka M. (2014). Endothelial connexin32 enhances angiogenesis by positively regulating tube formation and cell migration. Exp. Cell Res..

[41] Haefliger J.-A., Allagnat F., Hamard L., Le Gal L., Meda P., Nardelli-Haefliger D., Génot E., Alonso F. (2017). Targeting Cx40 (Connexin40) Expression or Function Reduces Angiogenesis in the Developing Mouse Retina. Arter. Thromb. Vasc. Biol..

[42] Alonso F., Domingos-Pereira S., Le Gal L., Derre L., Meda P., Jichlinski P., Nardelli-Haefliger D., Haefliger J.A. (2016). Targeting endothelial connexin40 inhibits tumor growth by reducing angiogenesis and improving vessel perfusion. Oncotarget.

[43] Fang J., Angelov S.N., Simon A.M., Burt J.M. (2011). Cx40 Is Required for, and Cx37 Limits, Postischemic Hindlimb Perfusion, Survival and Recovery. J. Vasc. Res..

[44] Buschmann I., Pries A., Styp-Rekowska B., Hillmeister P., Loufrani L., Henrion D., Shi Y., Duelsner A., Hoefer I., Gatzke N. (2010). Pulsatile shear and Gja5 modulate arterial identity and remodeling events during flow-driven arteriogenesis. Development.

[45] Nelson T.K., Sorgen P.L., Burt J.M. (2013). Carboxy terminus and poreforming domain properties specific to Cx37 are necessary for Cx37-mediated suppression of insulinoma cell proliferation. Am. J. Physiol. Physiol..

[46] Evans W.H., Boitano S. (2001). Connexin mimetic peptides: Specific inhibitors of gapjunctional intercellular communication. Biochem. Soc. Trans..

[47] Baudin B., Bruneel A., Bosselut N., Vaubourdolle M. (2007). A protocol for isolation and culture of human umbilical vein endothelial cells. Nat. Protoc..

[48] Tschumperle D., Deriche R. (2005). Vectorvalued image regularization with PDEs: A common framework for different applications. IEEE Trans. Pattern Anal. Mach. Intell..

[49] Carpentier G., Berndt S., Ferratge S., Rasband W., Cuendet M., Uzan G., Albanese P. (2020). Angiogenesis Analyzer for ImageJ—A comparative morphometric analysis of “Endothelial Tube Formation Assay” and “Fibrin Bead Assay” and “Fibrin Bead Assay”. Sci. Rep.

[50] Livak K.J., Schmittgen T.D. (2001). Analysis of relative gene expression data using real-time quantitative PCR and the 2(-Delta Delta C(T)). Methods.

